# Supplementary figures and images for: Beyond SOFA and APACHE II, Novel Risk Stratification Models Using Readily Available Biomarkers in Critical Care
Source: Diagnostics (Basel). 2025 Apr 28;15(9):1122. doi: 10.3390/diagnostics15091122 (PMC12071242; doi:10.3390/diagnostics15091122)

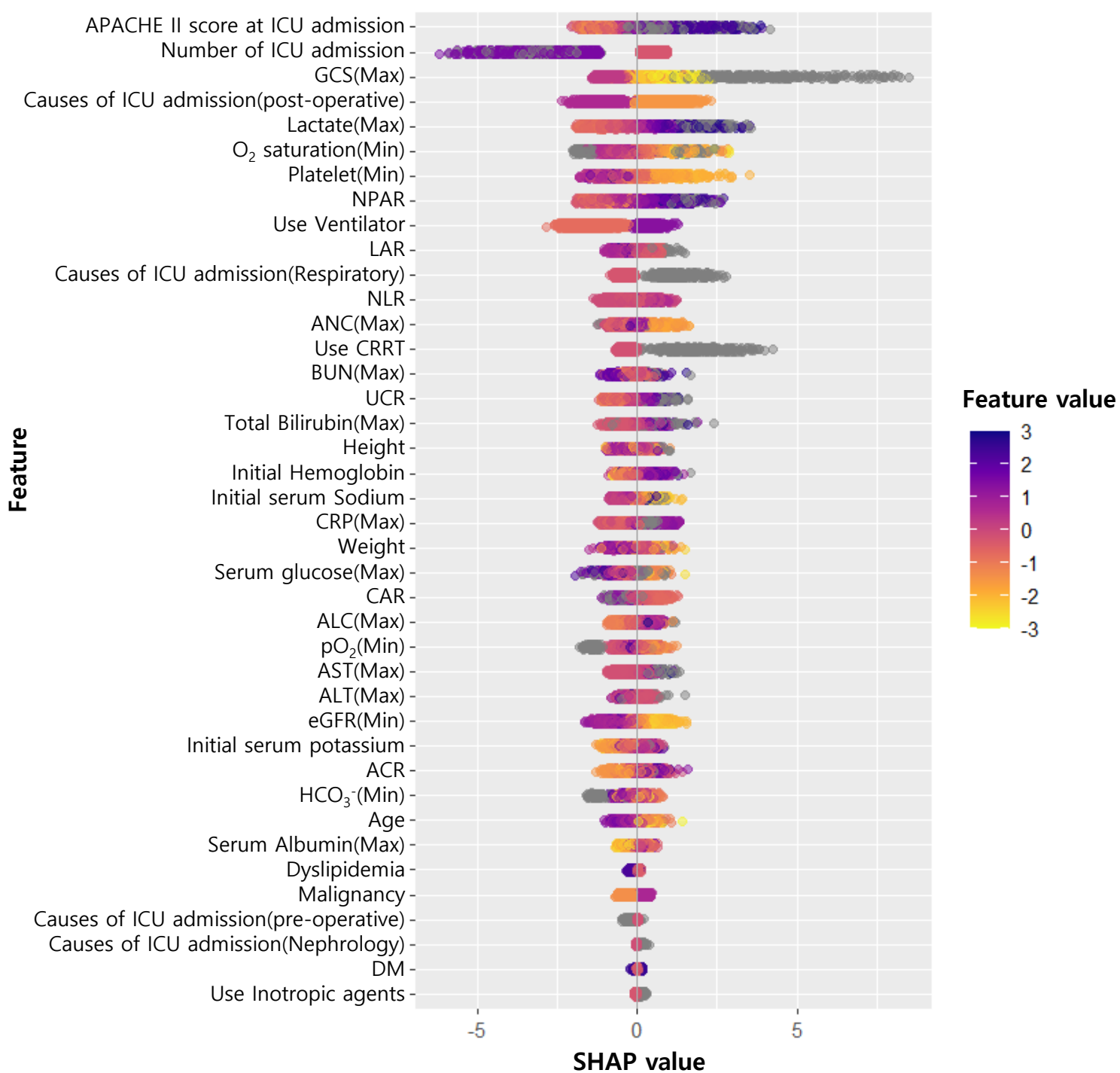

Supplement: Supplementary file 1 [file diagnostics-15-01122-s001.zip › diagnostics-3554797-supplementary.pdf]
